# Fear and Disgust of Spiders: Factors that Limit University Preservice Middle School Science Teachers

**DOI:** 10.3390/insects9010012

**Published:** 2018-01-29

**Authors:** Ron Wagler, Amy Wagler

**Affiliations:** 1 Department of Teacher Education: STEM Division, The University of Texas at El Paso, 500 West University Avenue, Education Building 601, El Paso, TX 79968, USA; 2 Department of Mathematical Sciences, The University of Texas at El Paso, 500 West University Avenue, Bell Hall 311, El Paso, TX 79968, USA; awagler2@utep.edu

**Keywords:** belief, disgust, fear, preservice middle school teachers, spiders

## Abstract

Spiders perform many essential ecological services, yet humans often experience negative emotions toward spiders. These emotions can lead to the avoidance of beneficial events. These emotions may affect beliefs about what should or should not be included in a science curriculum. This study investigated how activities with living spiders affected preservice middle school science teachers’ emotions and beliefs. Prior to the activities both groups (i.e., treatment and control) had moderate to extreme fear and disgust toward the spider. The teachers that participated in the spider activities (i.e., treatment group) had much lower levels of fear and disgust after performing the spider activities than the control group that did not participate in the spider activities. The control group continued to have elevated levels of fear and disgust toward the spider throughout the study. Before the spider activities neither group planned to incorporate information about spiders or information about the essential ecological services of spiders into their science classroom. After the treatment group participated in the spider activities, the teachers had definitive plans to teach their students about spiders and the essential ecological services that they provide. The control group remained unchanged and had no plans to teach this information to their students.

## 1. Introduction

More than 75% of all animal species are arthropods [1]. Within global ecosystems, arthropods perform many essential ecological services, such as the decomposition of dead organisms, breaking up soils by burrowing to facilitate plant root growth, participating in nutrient cycles that lead to soil fertility, maintaining an ecological balance by keeping other animal population densities at ecologically healthy levels, serving as food for other animals in complex food webs, pollinating plants, and a myriad of other scientific phenomenon that science is only now beginning to fully understand. Without arthropods performing these essential ecological services global ecosystems would quickly collapse and humans would become extinct [2]. Even though arthropods play an essential and beneficial role in worldwide ecosystems and humanity ultimately depends on ecosystems for its very survival, humans often experience emotions of fear and disgust toward arthropods [3,4].

Learning to fear other animals in an environment, from a biological perspective, is crucial to survival [5]. Similarly, the emotion of disgust is protective and benefits an organisms’ survival rate [6]. The emotions of fear and disgust serve as a behavioral mechanism that can influence humans to avoid pathogens, predators, and perceived dangerous events [3,6,7]. However, these emotions are often misplaced in modern society and can potentially lead to avoidance of beneficial events [8]. For example, a person may attempt to eradicate spiders from their garden that are providing biological control or avoid being in nature because they are fearful of spiders. In educational settings, it may be that the avoidance emotions of fear and disgust affect beliefs about what should or should not be included because of the science curriculum content the teacher is being asked to teach. Allowing for emotions of avoidance to affect curriculum choice can lead to an incomplete body of topics in a middle school science classroom.

To date no research has been conducted on the emotions university preservice middle school science teachers have toward arthropods and how these emotions impact their curriculum choices. This is an important group of teachers to evaluate because after elementary students learn about basic ecological concepts, such as food chains, these same students’ progress to the middle school science classroom where they should experience increasingly complex concepts associated with biodiversity, ecosystems, and ecosystem processes. If middle school science teachers are not equipped to teach these concepts to their students, these students will enter high school with a greatly reduced understanding of biodiversity, ecosystems and ecosystem processes. This article presents a research study that occurred in a university science methods course with preservice middle school science teachers that were non-science majors (i.e., not pursuing a bachelor’s degree in a scientific field such as biology). This randomized and controlled study investigated how activities with living spiders affected the university students’ avoidance emotions of fear and disgust toward spiders, their beliefs concerning the likelihood of incorporating information about spiders into their science classroom and their beliefs concerning the likelihood of incorporating information about the ecological services of spiders into their science classroom.

## 2. Theoretical Underpinnings of the Study

### 2.1. Human Fear and Human Disgust

The human emotions of fear and disgust are protective and produce the avoidance of potentially dangerous and disease-causing animals [9,10]. Specific animals evoke elevated levels of fear and disgust [6]. For example, spiders produce greater levels of fear and disgust in humans than beetles, bees/wasps, and butterflies/moths [3]. When humans find certain animals disgusting, they are often motivated to avoid that animal [7,9,11]. Correspondingly, fear can also motivate humans to avoid specific animals [3,6,12]. Even though the presence of fear and disgust toward spiders originates from a biologically based psychological mechanism, there are cultural factors that can influence the emotions of fear and disgust [13]. For example, the emotions of fear and disgust can be transferred from individuals in social settings and progress into patterns of avoidance that are specific to the type of organism [14].

### 2.2. Human Belief

Human belief is an estimation of the likelihood that the knowledge one has about an entity is correct or, alternatively, that an event or a state of affairs has or will occur [15]. Human belief can also be viewed as an estimation of subjective probability or the certainty that a proposition is true [16]. In the context of the current study, preservice middle school science teachers may hold specific beliefs about science curriculum content that can cause them to treat the topic in a negative manner [17]. Specifically, the preservice middle school science teachers may possess elevated levels of fear and disgust toward spiders, and these emotions may be negatively influencing their beliefs concerning the likelihood of incorporating information about spiders and the ecological services of spiders into their science classroom.

## 3. Literature Review

### 3.1. Spiders: A Brief Overview

Spiders are arthropods that have a chitinous exoskeleton, a segmented body, and jointed appendages [18]. Spiders are predatory arthropods that exist in almost all terrestrial and semi-terrestrial ecosystems on Earth. Spiders benefit humanity both indirectly and directly. For example, spiders benefit humans indirectly by reducing arthropod numbers in global environments. Many of these arthropod species, when present in elevated numbers, reduce agricultural crop yields that sustain humans and are vectors for human disease [19,20]. Spiders also indirectly benefit humans by serving as a food source for animals humans enjoy esthetically, such as monkeys, apes, and lemurs [21]. Lastly, spiders directly benefit humans by serving as a food source in some cultures [22].

### 3.2. Research Addressing the Use of Arthropods in Educational Settings with Preservice Teachers

Educational research studies addressing the use of arthropods in educational settings with preservice teachers are very limited. No studies have been conducted with inservice teachers. The vast majority of research in this area has been performed on preservice elementary teachers. One research study has been conducted assessing preservice middle school science teacher’s psychological tendencies and beliefs toward arthropods and other biodiverse animals. Wagler and Wagler assessed the relationship between preservice middle school science teacher characteristics, their attitude toward a specific animal, and their belief concerning the likelihood of incorporating information about that specific animal into their science classroom [23]. The participants self-reported their gender, age, number of college biological science courses taken, and their preference to teach biological science or physical science when they were an inservice middle school teacher. The participants were then shown thirty pictures of biodiverse animals (i.e., mammals, birds, reptiles, amphibians, arthropods, and other invertebrates). For each picture, the participants rated their attitude toward the animal shown. The participants then rated the likelihood, based on their attitude, of incorporating information about the animal shown into their science classroom.

The preservice middle school science teacher characteristics that positively increased the preservice middle school science teacher’s attitude or the likelihood of incorporating information about arthropods into their classroom were being a male, having taken one additional college biological science course or being older than 26 years of age. Specifically, if the preservice middle school science teacher was older than 26 or was a male, they had a more positive attitude toward arthropods. Furthermore, if the preservice middle school science teacher was older than 26 years of age and had taken an additional biological science course (with an animal biodiversity component) they were more likely to include information about arthropods in their science classroom but even then the inclusion of this information was minimal [23].

Because of the lack of research associated with preservice middle school science teachers, educational research is presented that addresses the use of arthropods in educational settings with preservice elementary teachers. The lack of research with preservice middle school science teachers in this area shows the need for more research with this population. Weinburgh assessed the impact a nine-week course intervention using mealworms (*Tenebrio obscurus*) had on the self-efficacy, content knowledge, and attitude of preservice elementary teachers [24]. Before the course intervention, the preservice elementary teachers showed minimal prior content knowledge about mealworms, were uncomfortable with the idea of having to teach with and about them, and expressed neutral attitudes toward mealworms upon first exposure to them. After the intervention, the preservice elementary teachers showed improved content knowledge about mealworms, improved self-efficacy about using mealworms in their teaching, and improved attitudes toward mealworms.

Wagler’s 2010 study found a strong statistically significant association between kindergarten through fourth grade (K-4) preservice elementary teacher’s attitudes toward a specific animal and their likelihood to exclude or include information about that animal in their science classroom [25]. Specifically, if a non-science major K-4 preservice elementary teacher had a positive attitude toward an animal, then they were much more likely to believe they would include information about that animal into their science classroom. Conversely, if a K-4 preservice elementary teacher had a negative attitude toward a specific animal they were much more likely to believe they would not include information about that animal into their science classroom. Based on these beliefs the K-4 science learning environment would be dominated by information about mammals. The teacher’s classroom would be void of any information about invertebrates (e.g., corals, sponges, crustaceans, worms, mollusks, arachnids and insects (Excluding the butterfly)), amphibians, and reptiles. This pioneering study provided the first empirical evidence that a preservice elementary teacher’s attitude toward a specific animal affected their belief about using information about that animal in their science classroom and showed this population has a strong aversion to the vast majority of invertebrate animals.

Wagler and Wagler’s 2011 study was the first study in the research field of arthropod education to utilize generalized latent variable modeling and employ a research design with randomly assigned treatment and control groups [26]. They found that preservice K-4 elementary teachers who had frequent direct contact with *Gromphadorhina portentosa* (the Madagascar hissing cockroach) in a positive educational setting during their preservice training program had their attitudes and likelihood of incorporating arthropod information into their science curriculum changed in a positive way toward the Madagascar hissing cockroach, but not toward other arthropods that they did not have contact with. The non-contact arthropods included a spider, dragonfly, crayfish, butterfly, scorpion, grasshopper, centipede, millipede, and lady beetle. Initially, the preservice elementary teachers possessed negative attitudes and low incorporation rates toward the Madagascar hissing cockroach, spider, crayfish, scorpion, grasshopper, centipede, and millipede, and positive attitudes and high incorporation rates toward the dragonfly, butterfly, and lady beetle. None of these attitudes and incorporation rates changed, except for the Madagascar hissing cockroach. This innovative research study established that in order to positively change preservice elementary teacher attitudes and incorporation rates toward a specific animal, direct frequent contact in a positive educational setting with that specific animal is needed.

Prior research studies had speculated that the external morphology of arthropods has a negative effect on human attitudes and emotions toward arthropods, but the topic had not been investigated empirically. In the first study of its kind, researchers investigated if the external morphology of an insect had a negative effect on kindergarten through sixth grade (K-6) preservice elementary teacher’s attitudes toward insects and beliefs concerning the likelihood of incorporating insect information into science education learning environments [27]. Non-science major K-6 preservice elementary teachers participated in the study and a randomized design with a control group was used. The K-6 preservice elementary teachers were shown color photographs of three insects (i.e., butterfly, lady beetle, and dragonfly) using a Microsoft PowerPoint presentation and were asked to rate their attitude toward the insects and beliefs concerning the likelihood of incorporating information about the insects into their elementary science classrooms. The treatment group was shown a picture of the larva and adult stage of the insect. The control group was only shown the adult stage of the insect. The study’s participants showed an overwhelming positive response to all of the adult insects, but, when shown the larval stages, they displayed overwhelming negative responses. Unique to the study was the finding that the external morphology of an insect is a causal factor that negatively affects preservice elementary teacher’s attitudes toward insects and beliefs concerning the likelihood of incorporating insect information into their elementary science classrooms.

Human negativity toward specific arthropods (e.g., cockroaches and spiders) has been well documented but the factors that contribute to this negativity have been elusive. In 2013, the first study to explore if knowledge of arthropod (i.e., spider and insect) carnivory and herbivory are possible casual factors that contribute to the overall negative tendencies preservice elementary teachers have toward most arthropods was conducted [28]. The study investigated the effect knowledge of arthropod carnivory and herbivory had on K-6 preservice elementary teacher arthropod attitude and belief concerning the likelihood of incorporating information about that specific arthropod (i.e., spider and insect) into their science classroom. A randomized design with a control group was utilized for the study. This study was the first to find that arthropod carnivory and herbivory are causal factors that strongly affect a preservice elementary teacher’s attitude and belief toward arthropods. When the participants of the study were made aware that an insect that they thought was a herbivore (i.e., lady beetle and dragonfly) was actually a carnivore, their attitude and likelihood of incorporation significantly declined. When the participants of the study were made aware that a spider they thought was a carnivore (i.e., the spider *Bagheera kiplingi*) was actually a herbivore, their attitude and likelihood of incorporation significantly increased.

Many teachers possess emotions of avoidance about science concepts, even when these very topics are an essential part of an effective science curriculum. Researchers investigated how emotions of avoidance affect curriculum choice in a science classroom and also evaluated a research-based social form of learning for changing emotions of avoidance toward a specific science topic (i.e., arachnids) for a population of preservice elementary teachers [29]. The study was performed using a research design with randomly assigned treatment and control groups. It was found that there was a strong invariant structural relationship between emotions of avoidance and beliefs about incorporation of science concepts about arachnids. An invariant structural relationship means that the relationships between the emotions of avoidance and science concepts were very similar across the treatment and control groups. However, participation in the arachnid learning activities decreased emotions of avoidance and increased beliefs about incorporation into a science classroom. The implications of these findings are that social forms of learning can change avoidance emotions and beliefs of teachers.

The fear of spiders in specific human groups is well documented, but little is known about other emotions and beliefs humans have toward most other arachnids. Because of the importance of arachnids to global ecosystems and the services that they provide to humanity, elementary children should learn about arachnids. However, prior research shows that preservice elementary teachers do not plan to include information about arachnids in their classrooms [26,28]. Researchers analyzed the effect a living arachnid workshop had on K-6 preservice elementary teachers’ emotions and beliefs toward living arachnids, and sought to see if the arachnid workshop could reduce the participants fear, perceived danger, and disgust toward arachnids and increase their likelihood of incorporating information about arachnids into their science classroom [30]. The study utilized a research design with randomly assigned control and treatment groups. Five living arachnids from five of the eleven extant arachnid orders were used in the study, which is the most biodiverse group of arachnids used in a study to assess the emotions and beliefs humans have toward arachnids. The study employs a longitudinal design (i.e., pretest, posttest 1, and posttest 2) with randomly assigned treatment and control groups thereby giving the researchers the ability to make casual claims and assess the effect of the intervention over a longer period of time. The treatment group (i.e., arachnid workshop participants) exhibited a steady and maintained decrease in the levels of fear, perceived danger, and disgust across the time points, while the control group (i.e., non-arachnid workshop participants) exhibited little change in these responses. A positive change in the likelihood of incorporation for each of the arachnids across time for the treatment group was found, while the control group showed little or no change in these responses across time.

## 4. Materials and Methods

### 4.1. Research Questions

1. Did the spider activities change the participants’ levels of fear toward spiders?
2. Did the spider activities change the participants’ levels of disgust toward spiders?
3. Did the spider activities change the participants’ beliefs concerning the likelihood of incorporating information about spiders into their science classroom?
4. Did the spider activities change the participants’ beliefs concerning the likelihood of incorporating information about the ecological services of spiders into their science classroom?

### 4.2. Study Participants

The studies participants consisted of university preservice middle school science teachers enrolled in a science methods course at a large urban Southwestern United States University with a predominantly Hispanic/Latino student population. All of the participants were non-science majors. The treatment and control group consisted of 214 participants. Of the 109 participants in the treatment group, 98 were female and 11 were male. The treatment participants mean age was 27.97 years and there were 102 Hispanic/Latino, 5 White, and 2 African-American. Of the 105 participants in the control group, 95 were female and 10 were male. The control participants mean age was 28.11 years and there were 99 Hispanic/Latino, 4 White, and 2 African-American. All university science methods course sections were randomized into a treatment or control group. The sections, and hence, treatment and control groups were homogenous with respect to gender, age, and ethnicity. Homogeneity tests comparing the ethnicity, age, and gender of the preservice teacher groups demonstrated that the treatment and control group were very similar with respect to these demographic characteristics.

### 4.3. Study Procedure

**Figure 1:**
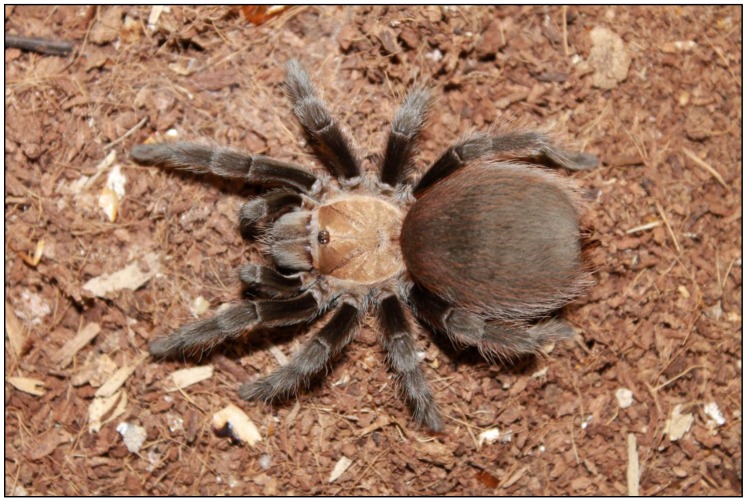
The Captive Bred *Aphonopelma* sp. Spider Used to Assess the Variables of the Study. (Photograph by Ron Wagler).

A pre/post randomized design with a control group was used for the study. A pretest was administered to all of the participants (treatment and control group) of the study at the beginning of the semester. The participants were shown a living captive bred spider (i.e., *Aphonopelma* sp.) (Figure 1) in a clear round plastic container (17.5 cm dia. × 8 cm H) with a secure clear plastic lid and 1 mm ventilation holes. Each participant was allowed to view the spider for up to 60 s from an approximate distance of 60 cm. The participants then rated their level of fear toward the spider (Likert scale: No fear (1) to Extreme fear (5)), level of disgust toward the spider (Likert scale: No disgust (1) to Extreme disgust (5)), likelihood of incorporating information about the spider into their science classroom (Likert scale: Extremely Unlikely (1) to Extremely Likely (4)), and likelihood of incorporating information about the ecological services of spiders into their science classroom (Likert scale: Extremely Unlikely (1) to Extremely Likely (4)). The treatment group then participated in the spider activities throughout the semester. The control group did not participate in the spider activities and learned nothing about spiders throughout the entire study. At the end of the semester after the treatment group participated in the spider activities, both of the groups completed the posttest by rating their level of fear toward the spider, level of disgust toward the spider, likelihood of incorporating information about the spider into their science classroom, and the likelihood of incorporating information about the ecological services of the spider into their science classroom, just as they had done for the pretest.

### 4.4. Overview of the Spider Activities

Only the treatment group performed the spider activities. The spider activities consisted of hands-on inquiry based middle school science activities from the articles “Teaching with Tarantulas” [31] and “A guide for Acquiring and Caring for Tarantulas Appropriate for the Middle School Science Classroom” [32]. These activities are from the peer reviewed National Science Teachers Association middle school science journal *Science Scope*. All of the spider activities emphasized basic biological information about spiders and information about the essential ecological services that spiders provide global ecosystems. Most of the activities emphasized the recent reduction in global spider numbers and one activity was completely focused on recent spiders that have gone extinct and an extant genus of spiders that is on the edge of extinction. The spider activities began after the pretest was administered at the beginning of the semester, continued throughout the semester and ended right before the posttest was administered at the end of the semester. The *Aphonopelma* sp. spider was chosen to assess the variables of the study. This species was chosen because they are safe for a classroom environment; frequently bred in captivity (so as to not remove further spider species from their indigenous habitat); easily purchased in the United States; and, easily incorporated into the studies hands-on inquiry based middle school spider activities. Furthermore, this spider and spider activities were chosen so that after the preservice teachers were trained in the use of the activities they could easily implement them in their own classroom.

### 4.5. Statistical Analysis

Cumulative multinomial logistic mixed models (CLMM) were used to model the probability that a single participant provided a rating from (1) to (5) on the Likert scale items regarding the levels of fear and disgust, as well as the likelihood of incorporating information about the spider and about the ecological services of the spider into their science classroom (Likert scale rating: (1) to (4)). Effects for the study participants within each section of the science methods course were included as random components in the CLMM. Log odds ratios summarize the model results. The reported log odds ratios compare the log of the odds of a one unit increase in the response (e.g., going from a (1) to a (2), (2) to a (3), etc.) for each of the endpoints of fear toward the spider, disgust toward the spider, likelihood of incorporating information about the spider into their science classroom (LIS), and likelihood of incorporating information about the ecological service of the spider into their science classroom (LIES) for the treatment versus control group. All analysis was performed in *R* [33] using the *ordinal* package [34]. The covariates used to predict the levels of fear, disgust, LIS and LIES were gender, ethnicity, an indicator variable for the treatment group, test time (pretest versus posttest), and all two-way and three-way interactions.

## 5. Results

Table 1 and Table 2 provide summary statistics about the primary endpoints (i.e., fear, disgust, LIS, and LIES). Table 3 provides parameter estimates for the log odds ratios and associated multiplicity adjusted *p*-values [35] resulting from the cumulative logit model for modeling the primary endpoints. In all of the analyses, there were significant two-way interactions between the variables indicating whether the participant was in the treatment group and the time point of the evaluation (Table 3).

## 6. Discussion

### 6.1. Findings

Research questions 1 and 2 assessed whether the spider activities changed the participants’ levels of fear and disgust toward the spider. Prior to the activities both groups had moderate to extreme fear and disgust toward the spider. The university students that participated in the spider activities (i.e., treatment group) had much lower levels of fear and disgust after performing the spider activities than the control group that did not participate in the spider activities. The control group continued to have elevated levels of fear and disgust toward the spider throughout the study (Table 1). Research questions 3 and 4 assessed if the spider activities changed the participants’ beliefs concerning the likelihood of incorporating information about spiders into their science classroom and beliefs concerning the likelihood of incorporating information about the ecological services of spiders into their science classroom. Before the spider activities, neither group planned to incorporate information about spiders or information about the ecological services of spiders into their science classroom. After the treatment group participated in the spider activities, the university students had definitive plans to teach their students about spiders and the essential ecological services that they provide. The control group remained unchanged and had no plans to teach this information to their students (Table 1). The ratings of fear, disgust, LIS, and LIES are correlated (Table 2), but current methodology does not allow for the joint modeling of these outcomes. In particular, note that in the upper portion of the table, it is evident that disgust is correlated with fear, LIES is positively correlated with LIS, and all other factors are negatively associated. The lower portion of the table provides the *p*-values associated with each estimated correlation. However, the complementary results of the separately modelled data suggest that the intervention (i.e., spider activities) affects these endpoints in a consistent fashion. The results in Table 3 indicate that for all of the endpoints (i.e., fear, disgust, LIS and LIES), there is a difference in the probability of a one unit increase in the Likert scale between treatment and control groups. In particular, for the ratings of fear and disgust, Table 3 results indicate that participants in the spider activities (i.e., treatment group) have decreased ratings of fear and disgust following the activities. In contrast, the control group (i.e., those that did not participate in the spider activities) had no change in their fear and disgust ratings for the spider. For the LIS and LIES endpoints, there was a significant increase in the LIS and LIES ratings for the participants who completed the spider activities but not for the control group.

### 6.2. Implications of the Study

When students do not learn about spiders and the ecological services that spiders provide, they have a very limited understanding about ecosystems and ecosystem processes [4]. Spiders are involved in a myriad of fascinating essential ecological processes that can greatly enrich the middle school science classroom experience but sadly this study shows that the emotions of fear and disgust are psychological barriers that keep many middle school teachers from planning to educate their students about spiders. This randomized and controlled study provides the first glimpse into the emotions and beliefs preservice middle school science teachers have toward spiders (or toward any arthropod), and how these emotions and beliefs impact their curriculum choices. This study provides very strong evidence that preservice middle school teachers have no plans to teach their students about spiders and the ecological services they provide, but through simple hands-on inquiry based educational interventions in their university courses these emotions can be changed and greatly increase the chances that they will teach this information to their middle school science students. These findings could not come at a more pertinent time as global spider numbers are being reduced and an ever-increasing number of spider species are on the edge of extinction or have recently become extinct [36], because of the environmentally destructive activities of humans associated with the ongoing 6th mass extinction [37]. Educating the next generation about the urgent need to protect the vanishing ecosystems that these spiders live in may very well be a component to maintaining long-term worldwide spider biodiversity. Education of this nature is needed to produce a future generation of concerned citizens, teachers, and scientists that can participate in the preservation of the global ecosystems that sustain these spiders. University instructors that teach courses for preservice science teachers and continuing education courses for inservice science teachers should begin to incorporate these simple, but effective, hands-on inquiry based spider activities (or similar activities) into their courses so that these teachers can include this information in their own science classrooms. Lastly, the findings of this study have a much broader application as these same simple activities can be integrated into any university course that has a biological or zoological component focused on animals that people often fear or find disgusting.

### 6.3. Future Research

On an even broader level the findings of this research study are relevant to university STEM (i.e., Science, Technology, Engineering, and Math) related courses where students (and possibly their university instructors) have negative avoidance emotions and beliefs toward other topics beyond spiders. There are many “hot button” topics, such as biological evolution, statistics, genetic engineering, human-induced climate change, etc. that are socially or politically sensitive that university students have negative emotions and beliefs toward that cause them to avoid these topics or not believe the current accepted findings in a given academic field. While the emotions and beliefs may differ for each topic, this study shows that the use of student centered hands-on inquiry based activities (as opposed to teacher centered classroom activities) is a highly effective way to address negative emotions and beliefs that lead to the avoidance of a given topic in an educational setting. Since this study has established an empirical relationship between emotions of avoidance and beliefs, the groundwork for examining these psychological relationships in future research studies can now be pursued. Preservice and inservice teachers possess avoidance emotions toward a variety of topics relating to mathematics and science [38]. This future research has promising outcomes for many other subject areas at the elementary, middle school, high school, and university level. Although the mechanism of these emotions may differ, the verification of this relationship in the current study suggests this association may hold true in other settings.

## 7. Conclusions

Spiders play an essential and beneficial role in worldwide ecosystems and should be included in middle school science curriculums. The incorporation of spider activities into the middle school science classroom provides an excellent way to develop a deeper understanding of ecosystems and ecosystem processes in middle school students. The current study shows that the emotions of fear and disgust are strong factors that inhibit preservice middle school science teachers from teaching their students about spiders and the ecological services spiders provide. The hands-on inquiry based spider activities used in this study are effective at changing these emotions and can greatly increase the chances that middle school science teachers will teach this information to their students. With an ever-increasing number of spider species on the edge of extinction, educational interventions of this nature are needed to produce a new generation that can participate in the preservation of worldwide ecosystems.

## Figures and Tables

**Table 1:** Group and Time Means (Standard Deviations) for Fear, Disgust, Likelihood of Incorporating Information about Spiders into their Science Classroom (LIS) and Likelihood of Incorporating Information about the Ecological Services of Spiders into their Science Classroom (LIES) Ratings.

Group	Time	Fear	Disgust	LIS	LIES
Control	Test One	3.74 (1.28)	3.71 (1.40)	2.11 (0.71)	2.10 (0.80)
Treatment	Test One	3.83 (1.25)	3.84 (1.31)	2.11 (0.70)	2.12 (0.77)
Control	Test Two	3.69 (1.29)	3.67 (1.40)	2.16 (0.72)	2.13 (0.82)
Treatment	Test Two	2.40 (0.93)	2.51 (0.77)	3.40 (0.65)	3.40 (0.56)

**Table 2:** Polychoric Correlations (Upper Triangular Region) and *p*-values (Lower Triangular Region) for Fear, Disgust, LIS and LIES.

	Fear	Disgust	LIS	LIES
Fear	1	0.320	−0.250	−0.238
Disgust	3.06 × 10^−9^	1	−0.191	−0.269
LIS	6.64 × 10^−24^	2.64 × 10^−32^	1	0.379
LIES	6.87 × 10^−13^	5.03 × 10^−36^	3.35 × 10^−41^	1

**Table 3:** Log Odds Ratio Estimates for Cumulative Logit Models Separately Fit for Fear toward Spiders, Disgust toward Spiders, Likelihood of Incorporating Information about Spiders into their Science Classroom (LIS) and Likelihood of Incorporating Information about the Ecological Services of Spiders into their Science Classroom (LIES) with Corresponding Pointwise *p*-values (Multiplicity Adjusted *p*-values).

	Fear	Fear *p*-Values	Disgust	Disgust *p*-Values	LIS	LIS *p*-Values	LIES	LIES *p*-Values
Male	−0.783	0.238 (0.821)	−0.383	0.623 (0.807)	0.527	0.478 (0.940)	0.156	0.730 (0.768)
Age	−0.006	0.799 (0.821)	−0.003	0.858 (0.858)	−0.003	0.911 (0.987)	−0.013	0.432 (0.768)
Hispanic	−0.790	0.600 (0.821)	−1.867	0.296 (0.807)	0.958	0.537 (0.940)	−0.284	0.768 (0.768)
White	−0.735	0.679 (0.821)	−1.053	0.623 (0.807)	0.410	0.825 (0.987)	−0.967	0.406 (0.768)
Treatment	0.103	0.821 (0.821)	0.209	0.672 (0.807)	0.008	0.987 (0.987)	0.111	0.738 (0.768)
Post	−0.172	0.559 (0.821)	−0.123	0.691 (0.807)	0.216	0.485 (0.940)	0.120	0.663 (0.768)
Trt*Post	−3.534	**0.000 (0.000)**	−3.499	**0.000 (0.000)**	6.270	**0.000 (0.000)**	3.996	**0.000 (0.000)**

Bolding of *p*-values denotes log odds ratio is statistically significant with and without multiplicity correction. Trt*Post is the interaction between the treatment and posttest time point.
